# Relationship Between Achilles Tendon Stiffness Using Myoton PRO and Translation Using a Tensile Testing Machine: A Biomechanical Study of a Porcine Model

**DOI:** 10.7759/cureus.49359

**Published:** 2023-11-24

**Authors:** Wataru Kurashina, Tsuneari Takahashi, Hideyuki Sasanuma, Akihiro Saitsu, Katsushi Takeshita

**Affiliations:** 1 Graduate School of Medicine, Jichi Medical University, Shimotsuke, JPN; 2 Department of Rehabilitation, Tochigi Medical Center Shimotsuga, Tochigi, JPN; 3 Department of Orthopaedic Surgery, Ishibashi General Hospital, Shimotsuke, JPN; 4 Department of Orthopaedics, Jichi Medical University, Shimotsuke, JPN

**Keywords:** objective assessment, stiffness, porcine, achilles tendon, biomechanics

## Abstract

Background

Achilles tendinopathy is a common ankle disorder in both the general population and athletes. This condition can alter the mechanical characteristics of the Achilles tendon (AT) by decreasing tendon stiffness. Achilles tendinopathy is primarily treated conservatively; however, few monitoring tools exist for evaluating the condition of the AT. The Myoton PRO (Myoton AS, Tallinn, Estonia) device is a handheld tool used to evaluate tissue stiffness. However, no basic studies have examined the validity of Myoton PRO for assessing the AT. This study aimed to assess the validity of Myoton PRO using animal ATs and to examine its clinical applicability.

Methods

We used 28 fresh porcine ankles and evaluated AT stiffness at the calcaneus insertion site (AT0) and 2.0 cm above the calcaneus (AT2) using Myoton PRO. We also measured changes in the AT length using a tensile testing machine during the cyclic loading test. We investigated the correlation between dynamic stiffness and length change. Furthermore, we assessed the difference in stiffness between AT0 and AT2.

Results

The dynamic stiffness was 717.6 ± 183.1 N/m at AT0 and 467.4 ± 152.3 N/m at AT2. The change in length during the cyclic loading test was 1.8 ± 0.7 mm. The correlation between dynamic stiffness and length change was as follows: AT0, r=-0.61; AT2, r=-0.64 (P<0.001). The dynamic stiffness at AT0 was significantly greater than that at AT2 (P<0.001).

Conclusions

AT assessment using Myoton PRO has potential clinical utility as an indicator of tissue stiffness.

## Introduction

Achilles tendinopathy is a common ankle disorder in both the general population and athletes [1]. Achilles tendinopathy is frequently observed in adults, with an incidence rate of 2.35 per 1,000. Notably, a relationship with sports activity has been recorded in 35% of the cases [2]. Achilles tendon (AT) injuries can be categorized based on the location of pain: insertional tendinopathy (20%-25%), midportion tendinopathy (55%-65%), and proximal musculotendinous junction (9%-25%) injuries [3].

Achilles tendinopathy is clinically diagnosed when patients present with a combination of localized pain, swelling of the AT, and loss of function [3,4]. Both subjective and objective orthopedic clinical tests have demonstrated significant diagnostic accuracy and capability for Achilles tendinopathy [5]. Imaging examinations, including magnetic resonance imaging (MRI) and ultrasound, have been used to rule out other injuries and provide additional clinical information [6,7]. Achilles tendinopathy can alter the mechanical, material, and morphological properties of the tendon structure, resulting in a decrease in stiffness and Young's modulus, along with an increase in tendon cross-sectional area and diameter [8,9]. Achilles tendinopathy is primarily treated conservatively, often through exercise therapy [10,11]. However, few optimal monitoring tools exist for evaluating the condition of the AT [12,13].

In recent years, the evaluation of AT stiffness using ultrasound elastography has become a valuable method [14]. However, the disadvantages of ultrasound elastography include differences in device performance and high cost, which make it difficult to use in clinical settings [15]. Another tool for evaluating stiffness, the Myoton PRO, correlates well with ultrasound elastography [16,17]. The Myoton PRO device is a handheld tool that can be used to evaluate tissue stiffness; however, no basic studies have examined its validity for assessing the AT. This study aimed to verify the validity of Myoton PRO using animal ATs and to examine its clinical applicability. We hypothesized that Myoton PRO correlates with a classical tensile testing machine.

## Materials and methods

Study design

Animal experiments were conducted in our institution’s biomechanics laboratory in accordance with the regulations of the Animal Care and Use Committee. The need for ethical approval from the committee was waived owing to the ex vivo nature of this study. We used porcine AT because of previous reports showing great similarities between pigs and humans in body weight, anatomy, histology, and immunology [18]. This study used 28 fresh porcine ankles (mean weight: 43.5 ± 4.2 kg; range: 38.1-51.6 kg). The tibia, fibula, talus, navicular, and cuboid bone were cut off, and the gastrocnemius and soleus muscles attached to the AT were carefully removed. Sutures were then placed proximal to the AT. The calcaneus was inserted into aluminum tubes with cement [19-20].

Dynamic stiffness using Myoton PRO

**Figure 1:**
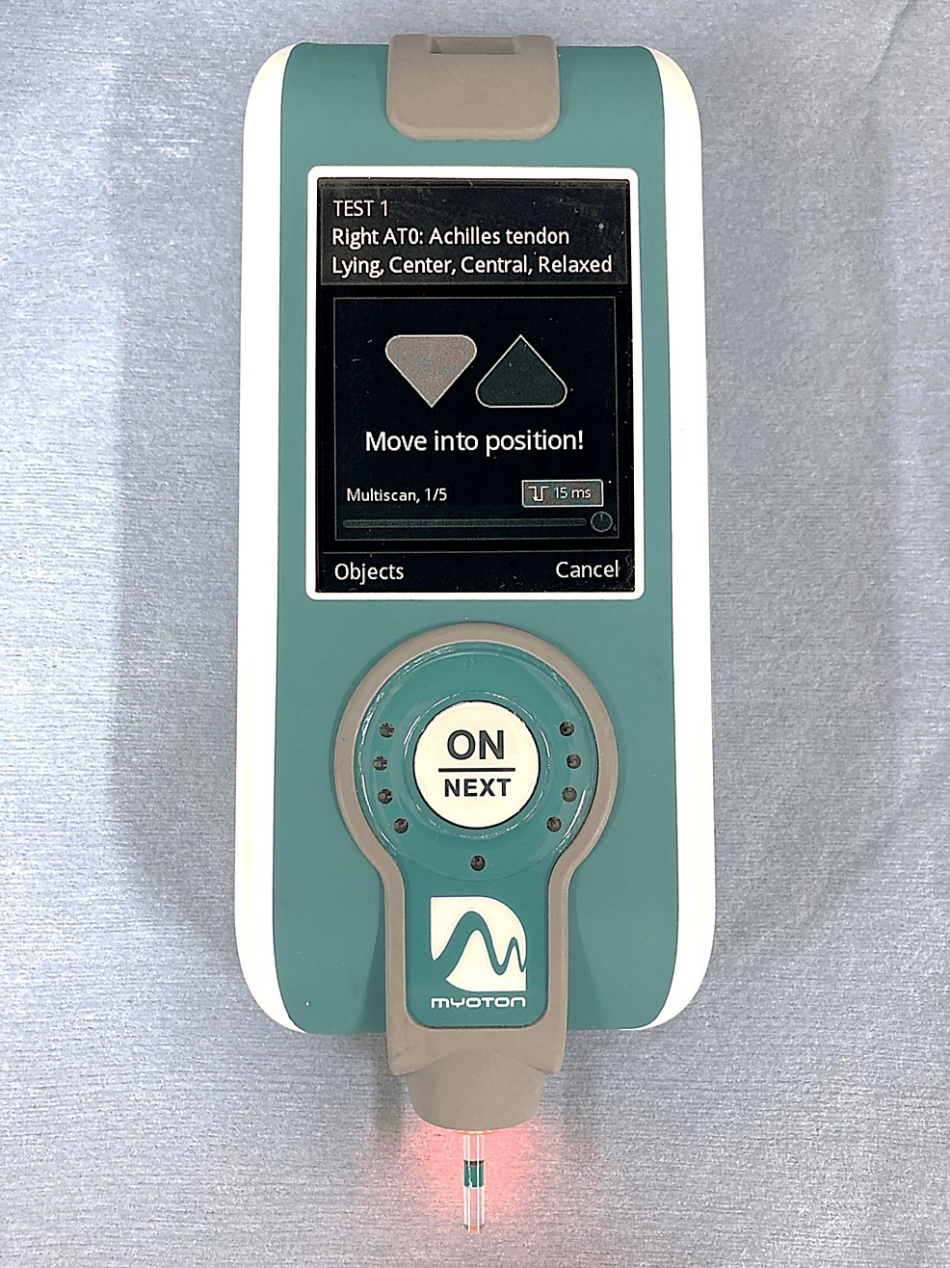
Photograph of the Myoton PRO device

Myoton PRO (Myoton AS, Tallinn, Estonia) is a handheld device that generates a mechanical impulse on the skin overlying a target structure [9] (Figure 1). The measurement method employed by Myoton PRO involves a mechanical impact released under constant prepressure (0.18 N) on the subcutaneous panniculus above the measured muscle or tendon. The oscillation of the tissue under the probe enables the calculation of the viscoelastic properties of the tissue. One parameter is dynamic stiffness, which is used to identify tissue characteristics [21]. This parameter can be calculated as the maximum acceleration of the oscillation and deformation of the tissue detected by the transducer (N/m) [22]. We evaluated AT stiffness at the calcaneus insertion site (AT0) and 2.0 cm above the calcaneus (AT2) using Myoton PRO. We obtained the average value of three measurements and assessed the intraobserver reliability using Myoton PRO.

Biomechanical testing using a tensile testing machine

**Figure 2:**
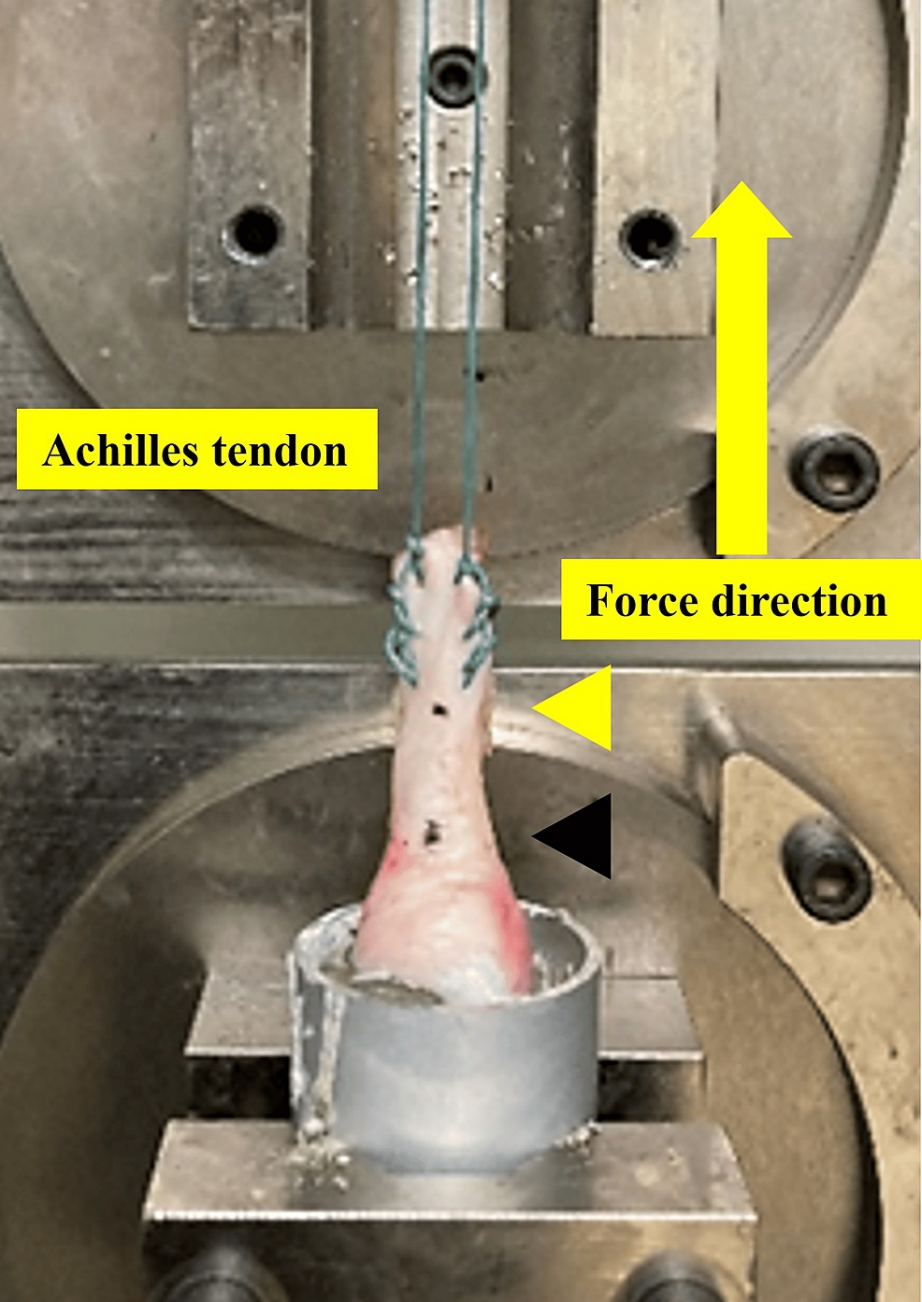
Biomechanical evaluation of Achilles tendon-calcaneus composite specimens The specimen was mounted on a tensile tester using a set of specially designed grips. A cyclic loading test was performed by applying a tensile load to the Achilles tendon, parallel to its long axis. The black arrowhead indicates the insertion of the Achilles tendon into the calcaneus, and the yellow arrowhead indicates the insertion point 2.0 cm above the calcaneus. These areas were measured using Myoton PRO.

**Figure 3:**
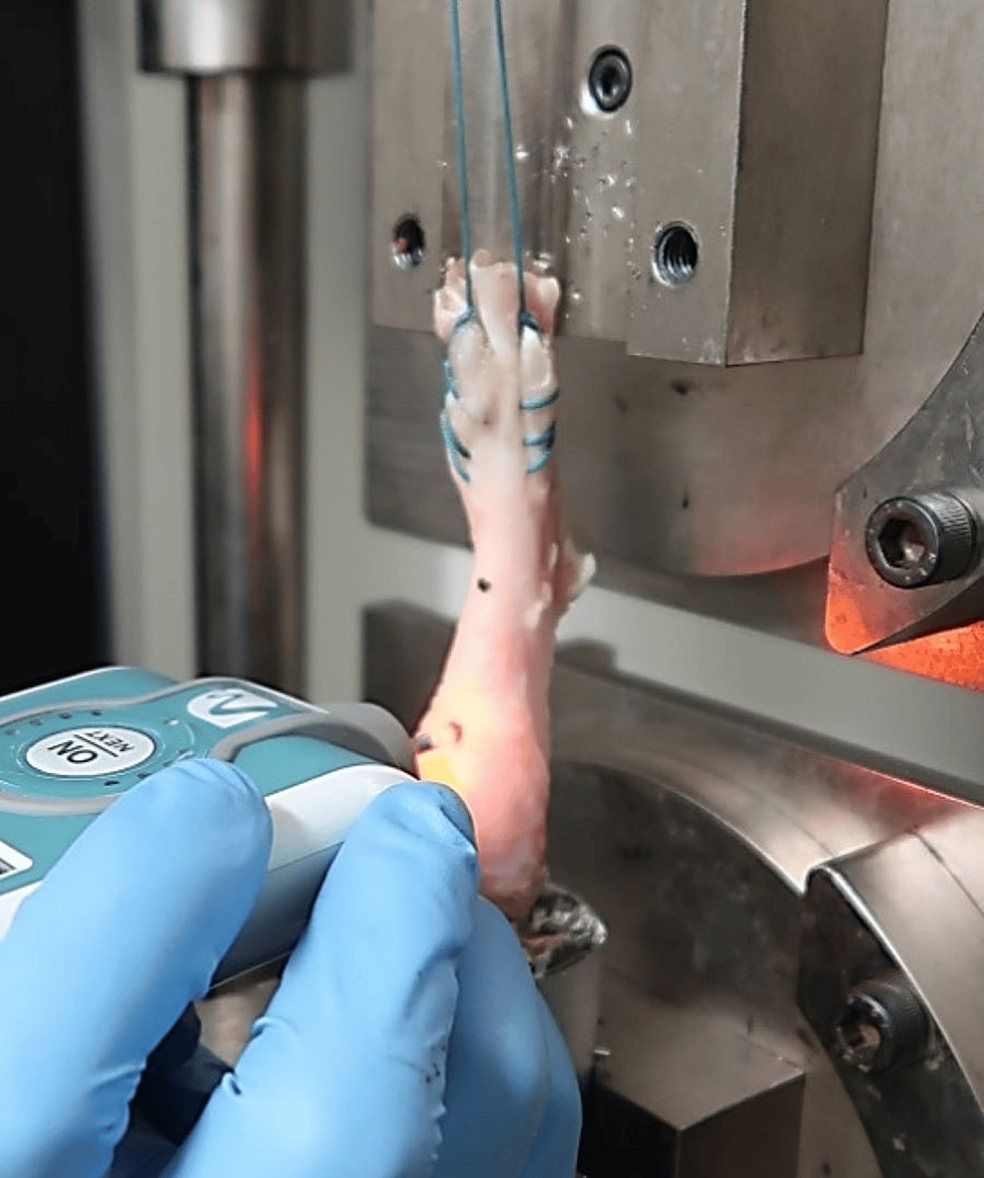
Procedure figure: Myoton PRO measurements at the insertion site of the Achilles tendon into the calcaneus

The prepared AT-calcaneus composite specimens were mounted on a tensile testing machine (Tensilon RTG 1250; Orientec Co., Tokyo, Japan) using specially designed grips (Figure 2). A saline solution was used to keep the structures moist during the experiment. Before testing, a static preload of 5 N was applied to the specimen, and then we evaluated the AT0 and AT2 stiffness using Myoton PRO (Figure 3). This assessment was followed by subjecting the specimen to 20 cycles of cyclic loading, ranging from 0 to 40 N, at a crosshead speed of 100 mm/min. This cyclic loading test was performed by applying tensile loading to the AT parallel to its long axis. The change in length during the cyclic loading was recorded. These conditions have been frequently used for measurements in previous studies using large-animal models [19-20].

Statistical analysis

An a priori power analysis was performed using G* Power 3.1 (Franz Paul, Kiel, Germany). We investigated the primary outcome of the correlation between the dynamic stiffness, measured using Myoton PRO, and the length change, measured using the tensile testing machine, using Pearson’s product-moment correlation coefficient. The sample size was calculated to require at least 26 samples, providing 80% power, with a Cohen effect size of 0.5 from a previous human AT study [15], to test the research hypotheses. As a secondary outcome, a paired t-test was performed to examine the differences in dynamic stiffness between AT0 and AT2. The intraclass correlation coefficient was used to evaluate the intraobserver reliability at AT0 and AT2. The reliability evaluation standard was judged to be high when the intraclass correlation coefficient was 0.75 or higher [23]. All data are presented as mean ± standard deviation. Statistical analyses were performed using Statistical Product and Service Solutions (SPSS) software, version 25.0 (IBM Corp., Armonk, NY, USA). Statistical significance was set at P<0.05.

## Results

**Figure 4:**
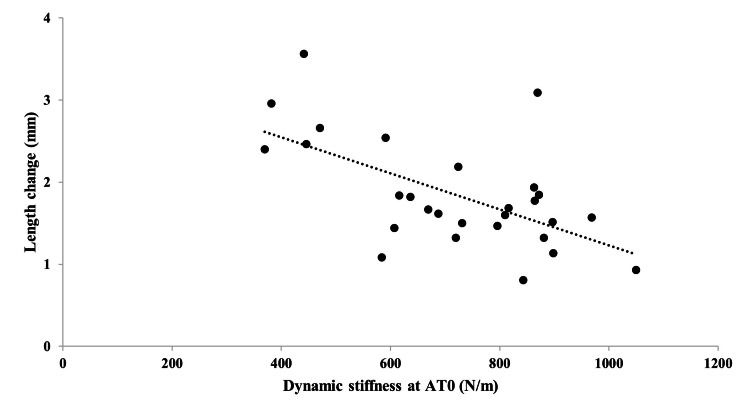
Correlation between dynamic stiffness at the Achilles tendon of the calcaneus insertion site (AT0) and length change

**Figure 5:**
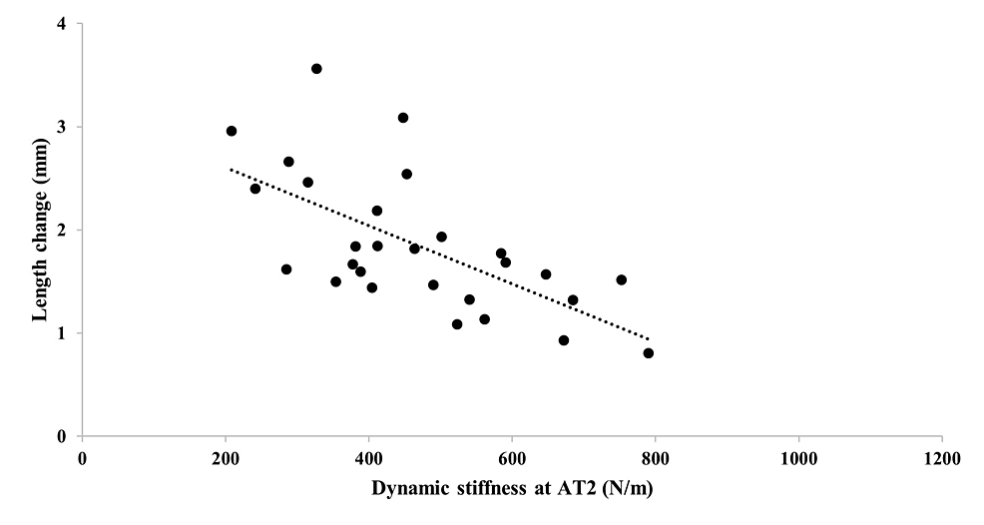
Correlation between dynamic stiffness at the Achilles tendon 2.0 cm above the calcaneus (AT2) and length change

The dynamic stiffness measured using Myoton PRO was as follows: AT0, 717.6 ± 183.1 N/m; AT2, 467.4 ± 152.3 N/m. The dynamic stiffness at AT0 was significantly greater than that at AT2 (P<0.001) (Table 1). The length change during cyclic loading was 1.8 ± 0.7 mm. The correlation between dynamic stiffness and length change during cyclic loading was moderately significant at AT0 (r=-0.61, 95% confidence interval (CI): -0.30 to -0.80)) and AT2 (r=-0.64, 95% CI: -0.36 to -0.83)) (all P<0.001) (Figures 4, 5). The intraobserver reliability was satisfactory (intraclass correlation coefficient: 1.3; AT0: 0.99 (95% CI: 0.98-1.00), AT2: 0.99 (95% CI: 0.98-1.00)).

**Table 1:** Dynamic stiffness(N/m) at AT0 and AT2 measured using Myoton PRO AT0, Achilles tendon of the calcaneus insertion site; AT2, Achilles tendon of the 2.0 cm above the calcaneus; CI, confidence interval Data are presented as mean ± standard deviation

Variables (n=28)	AT0	AT2	P values	95%CI
Dynamic stiffness (N/m)	717.6 ± 183.1	467.4 ± 152.3	< 0.001	202.3-298.2

## Discussion

In this study, the dynamic stiffness measured using Myoton PRO was significantly negatively correlated with the length change measured using a biomechanical machine at AT0 and AT2. Furthermore, a significant difference in tissue stiffness was observed between AT0 and AT2 when Myoton PRO was used. These results suggest that stiffness evaluation of the AT using Myoton PRO can not only estimate the amount of translation in the longitudinal direction of the tissue but also distinguish the stiffness of each site.

Few reports have explored the correlation between Myoton PRO and other AT devices. Feng et al. reported a moderate correlation between Myoton PRO and shear wave elastography in human AT [15]. The present large-animal study showed that dynamic stiffness, measured using Myoton PRO, exhibited a moderate correlation with length change, measured using biomechanical machines, at both AT0 and AT2, consistent with the findings of the human AT study [15]. Biomechanical testing using tensile testing machines on porcine anterior cruciate and medial collateral ligaments has been reported [19-20]. Significant length changes, as observed with the long-axis method, indicate greater translation of the tissue and softer tissue. Consequently, the dynamic stiffness values measured using Myoton PRO consistently appeared lower. To our knowledge, this is the first study to validate the Myoton PRO device using other devices in large animals.

A significant difference in stiffness was observed between AT0 and AT2 using Myoton PRO. Chang et al. measured the AT0 and AT6 cm portions and found differences in dynamic stiffness [24]. Huang et al. reported that AT stiffness decreased in a distal-to-proximal pattern [25]. Therefore, stiffness may differ depending on the AT measurement unit. Chimenti et al. reported that the insertion at the AT increased the concentration of cartilage matrix proteins, such as type 2 collagen and aggrecan, along with a change in the morphology of tendon cells [26]. The stiffness of the AT decreased with increasing distance from the calcaneus. The difference in dynamic stiffness between AT0 and AT2 in this study indicates the ability of Myoton PRO to accurately capture tissue stiffness at each site.

Achilles tendinopathy necessitates adjusting the load while checking the condition of the AT during rehabilitation at hospitals and athletic rehabilitation in sports field settings [13]. Until now, detecting abnormalities solely through MRI and ultrasound imaging and accurately tracking progress has been challenging [6]. In the present study, the Myoton PRO and biomechanical machine demonstrated a moderate correlation. Recently, Myoton PRO was reported to reduce AT stiffness in patients with Achilles tendinopathy [5,27]. Myoton PRO can also noninvasively and easily assess the condition of the AT. In the future, if this handheld tool becomes more versatile and intervention loading is examined while closely monitoring the AT, exercise therapy that considers the appropriate loading of the AT according to individual conditions may be provided.

This study had several limitations. First, we did not examine interobserver reliability. However, previous studies have reported that interobserver reliability is high to very high, similar to intraobserver reliability [9,24]. Because Myoton PRO accepts measurements with a coefficient of variation of less than 3% to delineate data reliability, all measurements exceeding this recommended value were repeated [5]. Myoton PRO exhibits high reproducibility in both intra- and inter-observer reliability, and intraobserver reliability alone is considered sufficiently reliable. Second, only two points of the AT were measured. Achilles tendinopathy has been reported to occur along the AT between 2.0 and 6.0 cm from its insertion point [28]. Therefore, specimens, including the gastrocnemius and soleus muscles, may be created and measured as a complex. Finally, because the specimens had normal ATs, the relationship with the disease was unknown. In the future, freeze-thaw or collagenase techniques should be used to verify the validity of in vivo tendon degeneration in animal models using Myoton PRO [29,30].

## Conclusions

Dynamic stiffness, measured using Myoton PRO, significantly correlated with length change, measured using a biomechanical machine, at AT0 and AT2. A significant difference in tissue stiffness was observed between AT0 and AT2 when Myoton PRO was used. Myoton PRO can be applied clinically to evaluate AT stiffness. Further research is required to monitor and manage tendon injuries in animal models and humans.
